# Regulation of Translation Initiation under Abiotic Stress Conditions in Plants: Is It a Conserved or Not so Conserved Process among Eukaryotes?

**DOI:** 10.1155/2012/406357

**Published:** 2012-04-23

**Authors:** Alfonso Muñoz, M. Mar Castellano

**Affiliations:** ^1^Departamento de Genética Molecular de Plantas, Centro Nacional de Biotecnología-CSIC, 28049 Madrid, Spain; ^2^Centro de Biotecnología y Genómica de Plantas, INIA-UPM, Campus de Montegancedo, 28223 Madrid, Spain

## Abstract

For years, the study of gene expression regulation of plants in response to stress conditions has been focused mainly on the analysis of transcriptional changes. However, the knowledge on translational regulation is very scarce in these organisms, despite in plants, as in the rest of the eukaryotes, translational regulation has been proven to play a pivotal role in the response to different stresses. Regulation of protein synthesis under abiotic stress was thought to be a conserved process, since, in general, both the translation factors and the translation process are basically similar in eukaryotes. However, this conservation is not so clear in plants as the knowledge of the mechanisms that control translation is very poor. Indeed, some of the basic regulators of translation initiation, well characterised in other systems, are still to be identified in plants. In this paper we will focus on both the regulation of different initiation factors and the mechanisms that cellular mRNAs use to bypass the translational repression established under abiotic stresses. For this purpose, we will review the knowledge from different eukaryotes but paying special attention to the information that has been recently published in plants.

## 1. Introduction

One of the main responses of cells to stress conditions involves partial or virtually total cessation of energetically consumptive processes normally vital to homeostasis, including transcription and protein synthesis. Translation consumes a substantial amount of cellular energy and, therefore, it is one of the main targets to be inhibited in response to most, if not all, types of cellular stresses. However, under conditions where global protein synthesis is severely compromised, some proteins are still synthesised as part of the mechanisms of cell survival, as these proteins are able to mitigate the damage caused by the stress and enable cells to tolerate the stressful conditions more effectively [1]. Appearance of abiotic stresses, as environmental conditions, is in many cases sudden. Therefore, a quick response to stress should be established to assure cell survival. In such a context, translational regulation of preexisting mRNAs provides a prompt and alternative way to control gene expression, as compared to other slower cellular processes such as mRNA transcription, processing, and transport to cytoplasm [2].

In animals and yeast, there are many known examples of global translational inhibition and preferential production of key proteins critical for survival under different abiotic insults [3–8]. This scenario also begins to be envisioned in plants where several studies demonstrate that general mRNA translation inhibition and selective translation of some mRNAs are key points in the adaptation process of plants to different abiotic stresses, including hypoxia, heat shock, water deficit, sucrose starvation, and saline stress [9]. Thus, in Arabidopsis seedlings subjected to oxygen deprivation, mRNAs coding proteins involved in glycolysis and alcoholic fermentation are efficiently translated, meanwhile the translation of other constitutively synthesised proteins is inhibited [10]. In a similar way, a decrease in the *de novo* protein synthesis has been demonstrated in *Brassica napus* seedlings after being subjected to heat shock for several hours. Under these conditions, in an opposite way to the proteins synthesised under normal conditions, only the translation of heat shock proteins is observed [11]. Furthermore, a reduction of protein synthesis with an increase in the synthesis of membrane proteins and of sulphur assimilation enzymes and transporters has been described in Arabidopsis-cultured cells subjected to sublethal cadmium stress [12]. In addition, the translational repression of specific components of the translation machinery and cell cycle-related mRNAs has been observed during sucrose starvation using the same system [13]. Other examples of rapid impairment of *de novo* protein synthesis by osmotic stress in Arabidopsis and rice have recently been published [14].

## 2. Initiation of Translation: Main Target of the Translation Regulation in Response to Abiotic Stress

To date, the different experiments carried out to unravel the translational phase regulated under stress conditions point to a regulation mainly at the initiation step. In eukaryotes, under physiological conditions, the vast majority of mRNAs initiate translation via a canonical cap-dependent mechanism that begins with the recognition by the eIF4E factor of the cap structure (7-methyl guanosine) placed at the 5′ end of the mRNAs to be translated. eIF4E interacts with eIF4G and with eIF4A, forming the cap binding complex called eIF4F. This complex allows the further recruitment of the preinitiation complex 43S, which consists of the small ribosomal subunit 40S, the ternary complex eIF2/GTP/tRNA_i_^met^ and the factors eIF3, eIF1 and eIF1A. The resulting pre-initiation complex scans the mRNAs in the 5′ → 3′ direction until an initiation codon is found. There the ribosomal subunit 60S is loaded and the elongation phase begins [15]. However, under abiotic stress conditions this canonical translation initiation is impeded by different mechanisms that affect mainly the activity of the initiation factors eIF2*α*, eIF4E, and eIF4A [1, 2, 5, 16–18].

## 3. Regulation of Translational Initiation Factors under Abiotic Stress

### 3.1. Translation Regulation by eIF2*α* Phosphorylation

**Figure 1:**
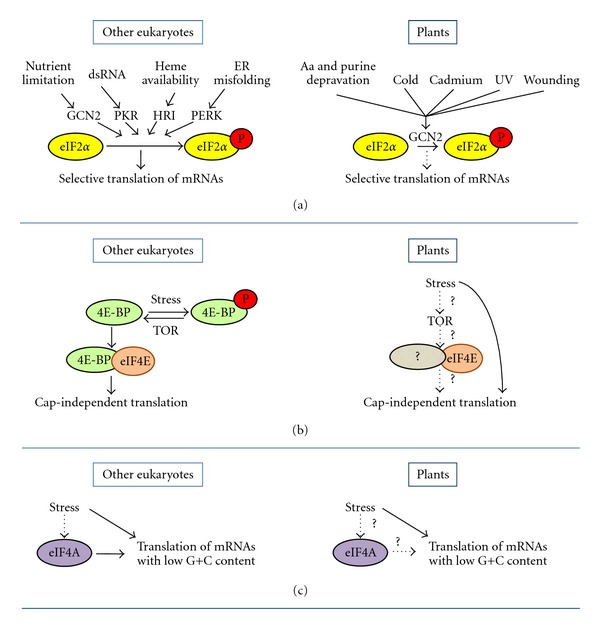
Regulation of translational initiation factors and transcript-differential translation under abiotic stress conditions. (a) Protein synthesis inhibition has been observed upon eIF2*α* phosphorylation both in plants and in other eukaryotes subjected to different abiotic stress conditions. In plants (right panel), in contrast to vertebrates (the case illustrated) (left panel), only the eIF2*α* kinase GCN2 has been described. In yeast and mammals eIF2*α* phosphorylation mediated by GCN2 promotes the selective translation of some mRNAs as *GCN4 *or *ATF4,* respectively; however, whether eIF2*α* phosphorylation leads to the stimulation of translation of specific mRNAs is unknown in plants. (b) In mammals the activity of eIF4E under abiotic stress is regulated by the eIF4E binding to hypophosphorylated forms of the 4E-BPs (left panel). Such binding promotes cap-dependent translation inhibition and the observation of cap-independent translation. Different evidences point out that plants can support cap-independent translation under abiotic stress conditions (right panel). However, the role of eIF4E and TOR in this process has to be elucidated. (c) Some abiotic stress conditions promote the selective translation of mRNAs with low G+C content in yeast and plants. In yeast (left panel) the involvement of eIF4A in this regulation has been described, although the exact mechanism regulating the activity of eIF4A is still unclear. In plants (right panel) the role of eIF4A in this process has to be determined.

In eukaryotes, one of the main mechanisms of translation inhibition in response to stress is the regulation of the subunit *α* of the eIF2 factor by phosphorylation. eIF2*α* phosphorylation is mediated by different kinases that are specifically activated in response to different stresses promoting the inhibition of translation by hindering the formation of the eIF2/GTP/tRNA_i_^met^ ternary complex [17]. eIF2*α* kinases and their activation by stress conditions are different among different eukaryotes. In vertebrates four different eIF2*α*-kinases, namely, GCN2, PERK, PKR and HRI that are activated by nutrient limitation [19], protein misfolding in the endoplasmic reticulum (ER) [20], virus infection [21], and heme group availability [22], respectively, have been described (Figure 1(a)). However, other eukaryotes have a different number of these enzymes. For instance, *Schizosaccharomyces pombe *has three eIF2*α* kinases (two distinct HRI and a GCN2), *Drosophila melanogaster *and *Caenorhabditis elegans *have only two (PERK and GCN2), and *Saccharomyces cerevisiae *has only one (GCN2) [23].

A strong inhibition of protein synthesis by eIF2*α* phosphorylation under different stress conditions has also been reported in plants, demonstrating that this mechanism of regulation of translation is conserved in these organisms [24]. Genome-wide searches for the presence of eIF2*α* kinases in Arabidopsis and rice suggest that higher plants only contain a GCN2-like eIF2*α* kinase [24]. In agreement with these *in silico* searches, so far only the eIF2*α* kinase GCN2 has been characterized in plants [24, 25], although some reports also suggest the controversial existence in plants of an eIF2*α* kinase with the biochemical properties of the mammalian PKR [26–29]. Arabidopsis GCN2 is activated under different stress conditions including amino acid and purine deprivation, cadmium, UV, cold shock, and wounding (Figure 1(a)), or in response to different hormones involved in the activation of defence response to insect herbivores [24, 25]. Although AtGCN2 activity is linked to a strong reduction in global protein synthesis under the aforementioned conditions, the activity of this enzyme does not account for the general inhibition of translation under all stresses in plants, as treatments using NaCl or H_2_O_2_ do not promote actively the phosphorylation of eIF2*α*. Moreover, results in Arabidopsis demonstrate that heat shock does not lead to eIF2*α* phosphorylation either, confirming previous results obtained in wheat [30]. Interestingly, heat shock causes a striking inhibition of protein synthesis in plants, suggesting that different mechanisms might be involved in the global protein synthesis inhibition observed under these conditions.

### 3.2. Translation Regulation by the Association of eIF4E with Interacting Proteins

The regulation of mammalian eIF4E under abiotic stress conditions is by far the mechanism that has been better studied. This regulation in mammals involves the interaction of eIF4E with the 4E-binding proteins (4E-BPs). 4E-BPs show the same conserved eIF4E-binding domain as eIF4G, so their action mechanism is based on their capability to compete out the eIF4G-eIF4E interaction, thereby inhibiting further recruitment of the ribosome to the mRNA “cap” structure. This mechanism is regulated by the phosphorylation status of 4E-BPs. Under physiological conditions, the TOR (target of rapamycin) kinase phosphorylates 4E-BPs, which turns 4E-BPs unable to interact with eIF4E. In response to different stresses, TOR is inhibited and 4E-BPs become dephosphorylated. This hypophosphorylation state increases the affinity of 4E-BPs for eIF4E, inhibiting cap-dependent translation and setting up a switch in the translational initiation mechanism from cap-dependent to cap-independent [18] (Figure 1(b)).

Regulation of eIF4E activity in budding yeast *S. cerevisiae *shares common features with that of mammals. In *S. cerevisiae* two functional homologs of the mammaliam 4E-BPs, p20 and EAP1, have been described [31, 32]. Both proteins block cap-dependent translation by interfering with the interaction of eIF4E with eIF4G, a mechanism analogous to that of the mammaliam 4E-BPs [31, 32]. In addition, TOR signalling pathway also plays a critical role in yeast, as in higher eukaryotes, in the modulation of translation initiation via regulation of eIF4E activity. Indeed, disruption of the *EAP1 *gene confers partial resistance to the growth-inhibitory properties of rapamycin, implicating EAP1 in the TOR signaling pathway controlling cap-dependent translation in *S. cerevisiae *[32].

Cap-independent translation has also been observed in plants subjected to both abiotic and biotic stresses (Figure 1(b)). In maize, two cellular mRNAs, the alcohol dehydrogenase *ADH1* and the heat shock protein *HSP101,* are translated in a cap-independent manner in oxygen-deprived roots [33] and during heat stress [34], respectively. These data, together with the fact that plant viruses use a cap-independent translation strategy to translate their mRNAs lacking the cap structure in the host cells [35], demonstrate that plant translational apparatus is able to support cap-independent translation under stress conditions. In addition, TOR also plays an important role in the regulation of protein synthesis in plants as RNAi reduction of *TOR* results in a strong inhibition of translation initiation in Arabidopsis, while *TOR*-overexpressing lines show an increase in translation initiation efficiency [36]. Moreover, in these lines the expression levels of *AtTOR* are correlated to the tolerance of Arabidopsis to osmotic stress indicating that AtTOR, possibly by its role in protein synthesis, modulates the response to abiotic stress conditions [36].

Regardless these striking parallelisms, the link between the role of TOR and the regulation of the eIF4E activity under abiotic stress in plants, if it exists, is far from being understood (Figure 1(b)). Indeed, no homolog of the 4E-BPs has been found in the plant genomes available to date. In spite of that, it has been described that the *β* subunit of the nascent polypeptide-associated complex (NAC) and the plant lipoxygenase 2 (AtLOX2) could putatively act as 4E-BP analogs since they interact with the Arabidopsis eIFiso4E in yeast two hybrid assays and these interactions can be displaced by the addition of AtIF4G* in vitro *[37, 38]. Moreover, AteIF4E has been proven to coimmunoprecipate with AtLOX2 from Arabidopsis extracts [38]. However, their role in the regulation of protein translation has not been demonstrated, as no evidences for changes in translation mediated by these proteins or for the regulation of their activities by TOR have been described either *in vitro* or *in vivo*.

### 3.3. Translation Regulation by eIF4A

Recently, new alternative mechanisms for the regulation of translation initiation under stress conditions which involve the regulation of the eIF4A RNA helicase have been discovered. A clear example is shown in yeast [5], where the authors demonstrated that glucose depletion causes a global translation inhibition due to a reduction in the amount of eIF4A bound to eIF4G. Concomitant with this reduction, changes in the levels of eIF3 associated to eIF4G are observed indicating that eIF4A could be required for the turnover in the association of eIF4G-eIF3 complex in a way that modulates translation initiation. Furthermore, the involvement of the regulation of eIF4A in translation in the response to lithium stress in *S. cerevisiae* has also been described [39] (Figure 1(c)).

As shown for the yeast eIF4A, plant eIF4A activity seems to be involved in the regulation of translation under abiotic stress in these organisms, as the overexpression of the pea DNA helicase 45, which seems to be the eIF4A ortholog, has been proven to confer high salinity tolerance in tobacco [40]. However, this observation should be further studied as the exact mechanism underlying this stress tolerance is not currently completely understood (Figure 1(c)).

## 4. Differential Translation of mRNAs in Response to Abiotic Stress Conditions

Under general translational inhibition conditions induced by abiotic stresses, some mRNAs involved in triggering stress responses are able to be selectively and efficiently translated. These transcripts have special characteristics that allow them to bypass specifically the different regulation points of translational inhibition. In this section we will focus on understanding the features that allow these mRNAs to circumvent downregulation of translation and we will deepen our knowledge in the information available in plants.

### 4.1. Differential Translation Mediated by eIF2*α* Regulation

Specific examples of mRNAs immune to eIF2*α* regulation under a variety of stress conditions as *GCN4* and *ATF4 *have been characterized in yeast [41] and mammals [42] (Figure 1(a)). Both mRNAs are able to be translated by a complex mechanism based on the fact that when eIF2*α* is phosphorylated and, therefore, the ternary complex is scarce, the scanning ribosome fails to initiate translation at upstream reading frames (uORFs), which are terminated in premature stop codons. In this case, scanning continues downstream towards the functional initiation codon allowing, with this long scanning, the enough time for ternary complex recruitment and, therefore, to promote the subsequent translation of the functional peptide [16, 41].

In plants, eIF2*α* phosphorylation causes a drastic inhibition of protein synthesis during amino acid starvation that is correlated with a partial inhibition of mRNA association to polysomes [24], demonstrating that, under eIF2*α* phosphorylation, there are some transcripts still able to be translated. However, at the moment, it is not known whether or not eIF2*α* phosphorylation leads to stimulation of translation of specific mRNAs, as reported for other systems (Figure 1(a)). In plants, no homolog to GCN4 transcription factor has been characterized and there is no evidence for the involvement of GCN2 in the transcriptional activation of Arabidopsis genes homologous to those regulated by GCN4 in yeast [25].

### 4.2. Differential Translation Mediated by IRESs and CITEs

In the late 1980s, the study of viral gene expression led to the discovery of the most studied alternative mode of translation initiation, the IRES-driven initiation. This mechanism allows the 40S ribosome to be directly recruited to sequences located within the 5′-UTR of viral RNAs called Internal Ribome Entry Sites (IRES) without the need of cap-recognition by eIF4E [43–45]. Since then, IRES activity has been described in an increasingly number of cellular transcripts including those coding for translation initiation factors, transcription factors, oncoproteins, growth factors, and homeotic and survival proteins. The presence of these cellular IRESs (cIRESs) allows the efficient translation of mRNAs under conditions, where cap-dependent initiation is inhibited or seriously compromised, as it is the case of abiotic stress or during physiological processes as mitosis, apoptosis, or cell differentiation [46, 47].

In plants, three cIRESs have been characterized to support cap-independent translation *in vitro*. These cIRESs have been found within the 5′-leader sequences of the mRNAs coding for the Arabidopsis ribosomal protein S18 subunit C (RPS18C) [48], the maize heat shock protein 101 (HSP101) [34], and the maize alcohol dehydrogenase (ADH1) [33]. Two of these mRNAs, the *HSP101* and the *ADH1* mRNAs, are efficiently translated under heat shock and under hypoxia, respectively [33, 34], suggesting an important role of cIRESs in the mechanism for selective translation under abiotic stress in plants. Indeed, the 5′-leader of* ADH1* was able to provide efficient translation of a reporter gene *in vivo* in *Nicotiana benthamiana* cells both under oxygen shortage and heat shock, while translation of the same construct lacking this sequence was significantly reduced [33]. Although promising, the examples of known plant cIRESs are scarce and, therefore, whether the use of cIRESs as translational enhancers of specific cellular mRNAs under abiotic stress is a generalized mechanism in these organisms remains still an open question.

For years the presence of cIRESs has been considered the only possible mechanism underlying cap-independent translation of cellular mRNAs. Interestingly, new mechanisms of cap-independent translation have been proposed to explain the translation observed under conditions where eIF4E activity is reduced [49, 50]. One of them is the translation of the mouse *HSP70* mRNA under heat stress conditions [4]. In this paper, Sun and collaborators demonstrate that the *HSP70 *5′-UTR is able to drive the translation of reporter genes under cap-independent conditions. However, the same sequence is unable to maintain cap-independent translation when placed in the intercistronic region of a bicistronic construct, ruling out the presence of an IRES within the sequence. Examples of such sequences have been described within plant viral mRNAs. The mRNAs of a large portion of all plant viruses lack the cap structure and, therefore, are forced to be translated in a cap-independent manner. To do so, in addition to viral IRESs, they use special elements termed cap-independent translational enhancers (CITEs). CITEs are able to recruit eIF4E and eIF4G cognates, or directly the 40S ribosomal subunit to the proximity to the AUG initiation codon, licensing in such a way the mRNA to initiate translation in a cap-independent manner [35, 51]. Although the existence of CITE-like elements is still considered exclusive of plant viral mRNAs, it would not be surprising if such elements are also discovered in plant cellular mRNAs. Cellular CITE-like elements, if present, might provide an alternative to cIRESs to drive translation of plant mRNAs [33].

Differential translation of some mRNAs under certain abiotic conditions could also be explained by the binding of specific RNA binding proteins to certain sequences within the mRNAs, acting as cap-dependent translational enhancing factors and cap-dependent enhancers, respectively. Most abiotic stress conditions reduce cap-dependent initiation and, therefore, enhancers acting synergistically with the cap could increase selectively the translational rate of those transcripts containing them. A good example of cap-dependent enhancing factors is the protein disulfide isomerase (PDI) that is a key regulator of insulin translation in response to glucose in mammals [52]. PDI is able to bind specifically to glucose responsive mRNAs under glucose stimulation and recruits the poly(A)-binding protein (PABP) to unknown enhancer elements in their 5′-UTR. Although how PABP binding could increase translation of such mRNAs is still unknown, it is reasonable to think that it is by the interaction of PABP with eIF4G. Cap-dependent enhancers of translation in plant viruses have also been described [53–55], being one of the better known examples the Ω sequence found in plant tobacco mosaic virus (TMV) [56]. This sequence is recognized by the HSP101 that, in turn and through its interaction with the Ω sequence, recruits eIF4G subunit to the 5′-UTR of the viral RNA [55]. The Ω sequence has been used to promote translation of cellular mRNAs enhancing both cap-dependent and cap-independent translation of the downstream gene by 2–10-fold. Therefore, these enhancers of cap-dependent translation could facilitate cap-dependent translation and even sustain some cap-independent translation under low eIF4E activity. If these kind of enhancers are also found in plant cellular mRNAs is a question that remains unanswered but that should be studied.

### 4.3. Differential Translation Mediated by eIF4A Regulation

Sequence analysis of polysome-bound mRNAs during glucose starvation in yeast, where a reduction of eIF4A association within the initiation complexes was observed, demonstrates that a common feature of these mRNAs is the low G+C content immediately upstream of the AUG [5]. These results suggest that the specific translation of mRNAs with low secondary structure could be selectively promoted under low eIF4A activity (Figure 1(c)). However, other alternative explanations cannot be fully excluded as, for example, the activation of IRES-driven translation of unstructured mRNAs by low level of helicase activity [6] or the possibility that other RNA helicases, with substrate preference for poorly structured mRNAs, may substitute the function of eIF4A. In a similar way, a study in Arabidopsis demonstrated that ribosome loading of mRNAs with high G+C content is differentially reduced under mild dehydration conditions [57]. These results may reflect, as in the previous case, a higher requirement for RNA helicase activity to initiate translation under stress in plants and may point to a low mRNA G+C content as a mechanism to bypass the restrain in eIF4A activity under abiotic stress (Figure 1(c)).

## 5. Unique Features of Regulation of Translation Initiation in Plants

It is well known that plants have unique translational characteristics as the existence, in addition to the canonical eIF4E and eIF4G factors, of IF(iso)4E and eIF(iso)4G isoforms. In Arabidopsis, one eIF(iso)4E and two eIF(iso)4Gs have been described; however, the number of these isoforms varies between plant species. eIF(iso)4E and eIF(iso)4G isoforms interact specifically between them to form eIF(iso)4F complexes [58]. The ability of the eIF(iso)4F complexes to support translation initiation of specific mRNAs has been proven different to that of the eIF4F complexes, suggesting that certain mRNA features allow different transcripts to interact preferentially with either complexes [59, 60]. Indeed, Lellis and coworkers have recently demonstrated that the double-mutant in the two Arabidopsis eIF(iso)4G factors displays strong phenotypes in growth and development, which, in the apparent absence of general protein synthesis inhibition, could be caused by the selective translation of specific genes [61]. Moreover, in maize it has been demonstrated that eIF(iso)4E is particularly required for the translation of stored mRNAs from dry seeds, and that eIF4E is unable to fully replace this eIF(iso)4E function [62]. If eIF4F and eIF(iso)4F complexes regulate translation of different sets of mRNAs, this would mean a plant-specific layer of gene expression regulation that is worth studying in depth.

## 6. Conclusion

The conservation of mechanisms to globally inhibit protein synthesis concomitant to mRNA translation reprogramming under different stresses points out to the fundamental importance of translation regulation during the response to abiotic stresses in all eukaryotes. Although we already know that there are multiple parallel mechanisms across eukaryotes that modulate translation under abiotic stresses, we are still far away from understanding completely this regulation, as new alternative mechanisms taking part in this regulation are still being described. In plants, the study of translational regulation under stress is still in its infancy, and some of the most conserved regulators have not been found in these organisms yet. A considerable effort should be done in this respect, since understanding how plants respond to environmental conditions can only be fulfilled by a complete knowledge of how translation is regulated.
